# Supplementary material for: Unbiased screening identifies regulators of cell-cell adhesion and treatment options in pemphigus
Source: Nat Commun. 2024 Sep 14;15:8044. doi: 10.1038/s41467-024-51747-2 (PMC11399147; doi:10.1038/s41467-024-51747-2)
Supplement: Supplementary file 1 — Supplementary Information [file 41467_2024_51747_MOESM1_ESM.pdf]

a

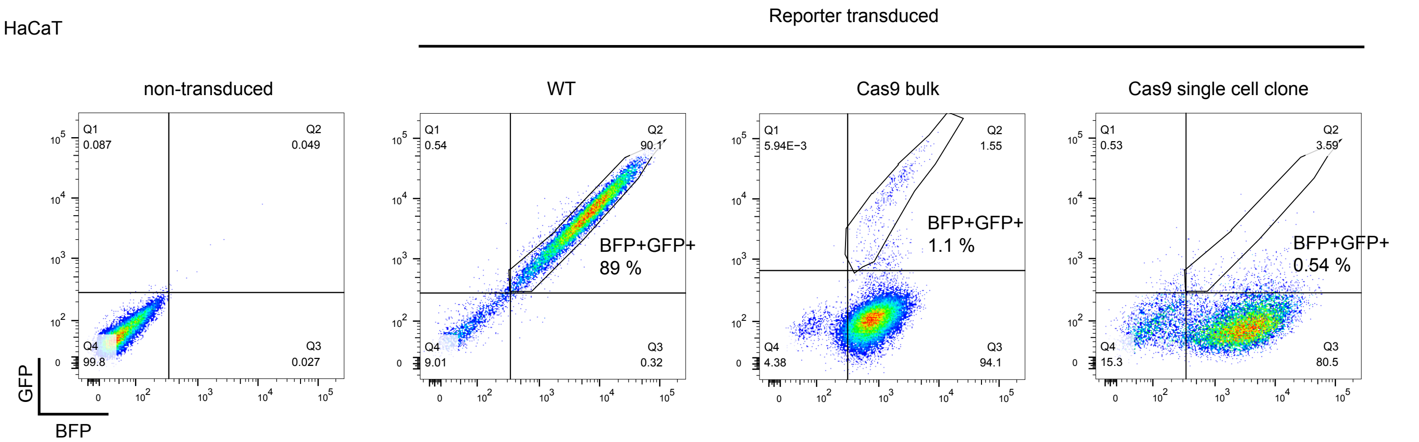

b

GO-ID	p-value	corr p-value	Description	Genes in test set
10467	1.63E-05	8.49E-03	gene expression	PSENEN,ABT1,YARS,ADAR,DDX51,EGFR,HCFC1,MSL1,SYNCRIP,TAF6L,KLF5,SPCS1,EIF3L,TCEB2,EIF3E
16043	1.71E-05	8.49E-03	cellular component organization	HDAC3,JUP,PDPK1,AP2A1,AP3B1,LRP3,ARPC4,PTPN11,RBPJ,DYNLL1,ATP2C1,EGFR,MSL1,TAF6L,ATP5B,KLF5,AP2S1,TCEB2,GOPC,GRB2,NF2,ELANE
16192	3.05E-05	9.80E-03	vesicle-mediated transport	ATP5B,AP2S1,AP3D1,AP2A1,VPS35,AP3B1,LRP3,GOPC,GRB2,ELANE
16044	4.83E-05	9.80E-03	cellular membrane organization	ATP5B,AP2S1,AP2A1,AP3B1,LRP3,GRB2,EGFR,ELANE
61024	4.92E-05	9.80E-03	membrane organization	ATP5B,AP2S1,AP2A1,AP3B1,LRP3,GRB2,EGFR,ELANE
10324	1.17E-04	1.67E-02	membrane invagination	ATP5B,AP2A1,AP3B1,LRP3,GRB2,ELANE
6897	1.17E-04	1.67E-02	endocytosis	ATP5B,AP2A1,AP3B1,LRP3,GRB2,ELANE
48661	1.38E-04	1.71E-02	positive regulation of smooth muscle cell proliferation	RBPJ,EGFR,ELANE
33157	1.64E-04	1.81E-02	regulation of intracellular protein transport	JUP,PTPN11,GOPC,EGFR
70201	1.88E-04	1.88E-02	regulation of establishment of protein localization	JUP,PDPK1,PTPN11,GOPC,EGFR
6139	2.48E-04	2.24E-02	nucleobase, nucleoside, nucleotide and nucleic acid metabolic process	ABT1,YARS,UHRF1,REV1,ADAR,DDX51,RBPJ,ATP2C1,HCFC1,MSL1,SYNCRIP,TAF6L,ATP5B,KLF5,TCEB2,EIF3E

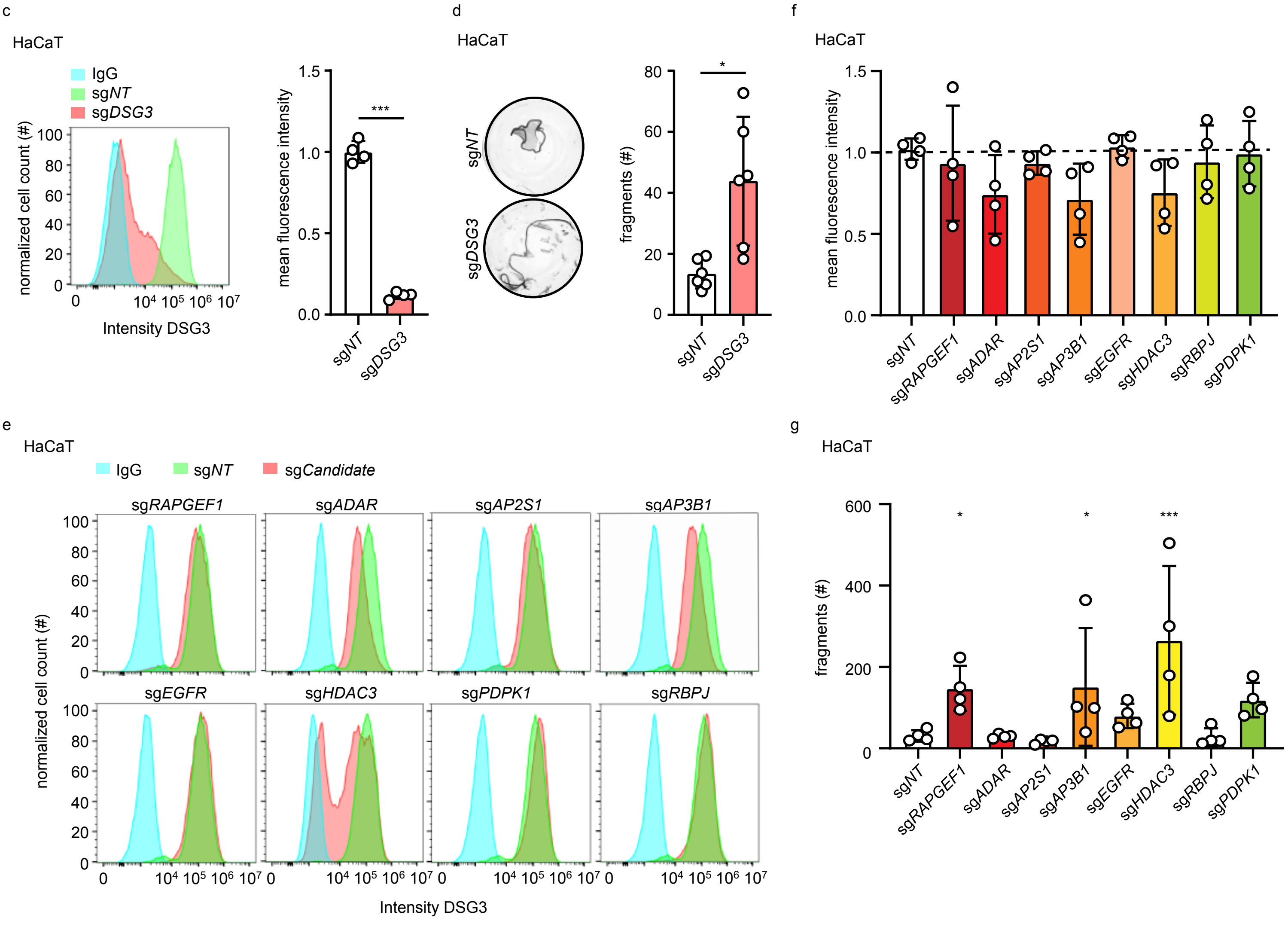

Supplemental Figure 1: a) Cas9 editing efficiency measured by flow cytometry of HaCaT cells transduced with pKLV2-U6gRNA5(gGFP)-PGKBFP2AGFP-W. The BFP+GFP- population corresponds to transduced and edited cells. Representative FACS plots are shown. b) GO-biological processes analysis of positive DSG3-regulators using BinGO in Cytoscape 3.10.0. The 10 groups with the most significant p-values are shown. c) Flow cytometry analysis of HaCaT cells stained with DSG3 antibodies or IgG as control. HaCaT cells were transduced with sg*NT* or sg*DSG3*. A representative histogram is shown and the mean fluorescence intensity (n=4, p<0.0001) is displayed. d) Fragment numbers counted in dispase-based dissociation assays of HaCaT cells stably transduced with sg*NT* or sg*DSG3* (n=5, p=0.0157). e) Flow cytometry analysis of HaCaT cells stained with DSG3 antibodies or IgG as control. HaCaT cells were transduced with sg*NT* or sg*RNA* directed against the indicated candidate gene. A representative histogram is shown and f) the mean fluorescence intensity (n=4) is displayed. g) Fragment numbers counted in dispase-based dissociation assays of HaCaT cells stably transduced with sg*NT* or sg*RNA* directed against the indicated candidate gene (n=4, sg*NT* vs sg*RAPGEF1* p=0.0481, sg*NT* vs sg*AP3B1* p=0.0376, sg*NT* vs sg*HDAC3,* p=0.0004). Values expressed as mean with standard deviation (mean+/-SD). One n represents one biological replicate. Source data are provided as a Source Data file. Experiments c) and d) were analyzed with two-sided student`s t-test; f) and g) were analyzed with One-way-ANOVA, SIDAK correction. p<0.05 *; p<0.01 **; p<0.001 ***

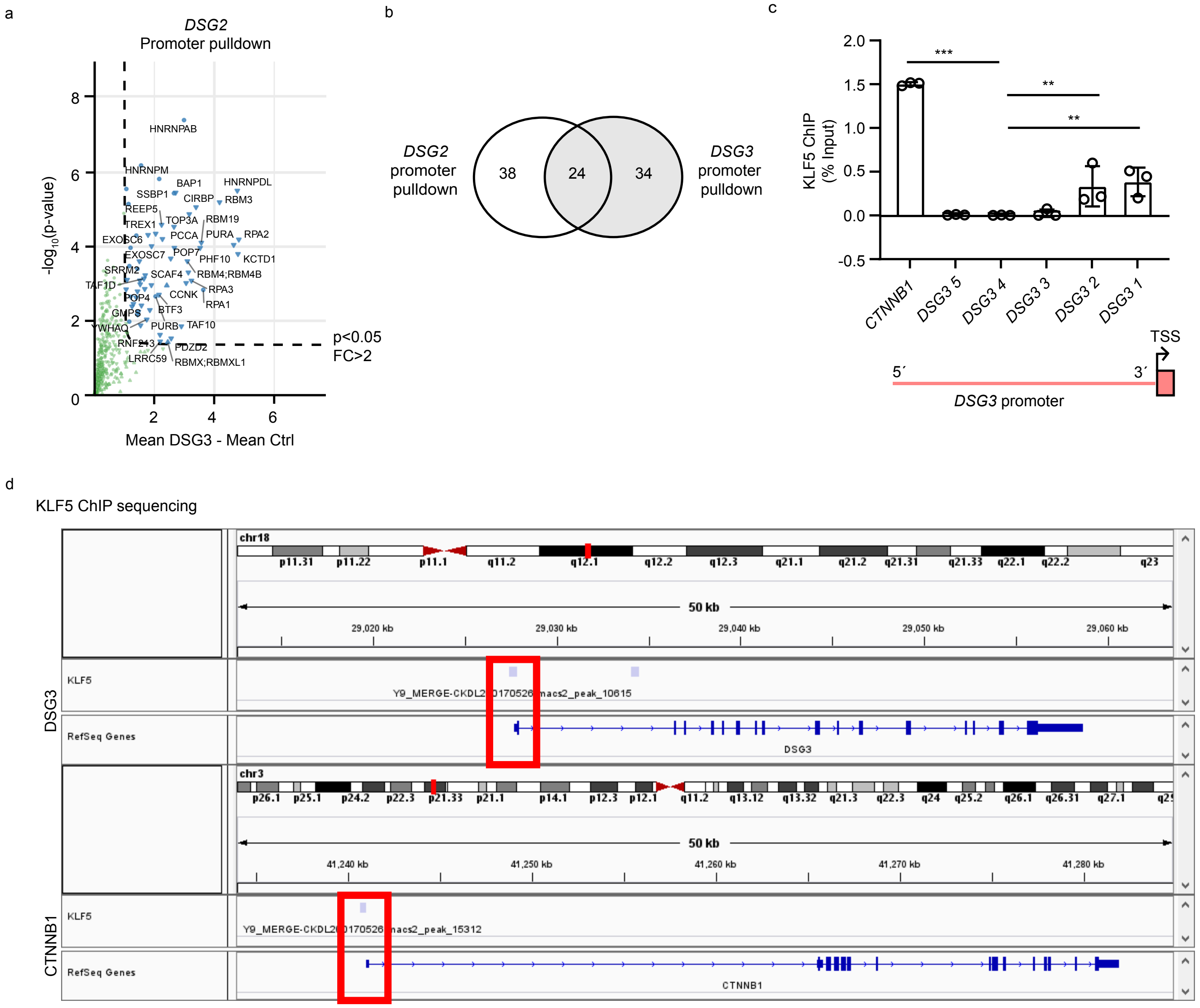

Supplemental Figure 2: a) Scatterblot of genes enriched at the DSG2 promoter blotted according to fold change and p-value. b) Overlap of proteins binding to the DSG2 promoter identified by promoter pulldown and proteins binding to the DSG3 promoter identified by promoter pulldown. c) ChIP-qPCR analysis using KLF5 antibodies and primers binding to CTNNB1 or different locations of the DSG3 promoter (n=3, CTNNB1 vs DSG3_4 p<0.001, DSG3_1 vs DSG3_4 p=0.0088, DSG3_2 vs DSG3_4 p=0.0028). d) ChIP-sequencing analysis of HaCaT cells (GEO number: GSE168600) with KLF5 antibodies, significant peaks on the DSG3 and CTNNB1 genes are displayed in the IGV browser. Values expressed as mean with standard deviation (mean+/-SD). One n represents one biological replicate. Source data are provided as a Source Data file. Experiment c) was analyzed with One-way-ANOVA, SIDAK correction. p<0.01 **; p<0.001 ***

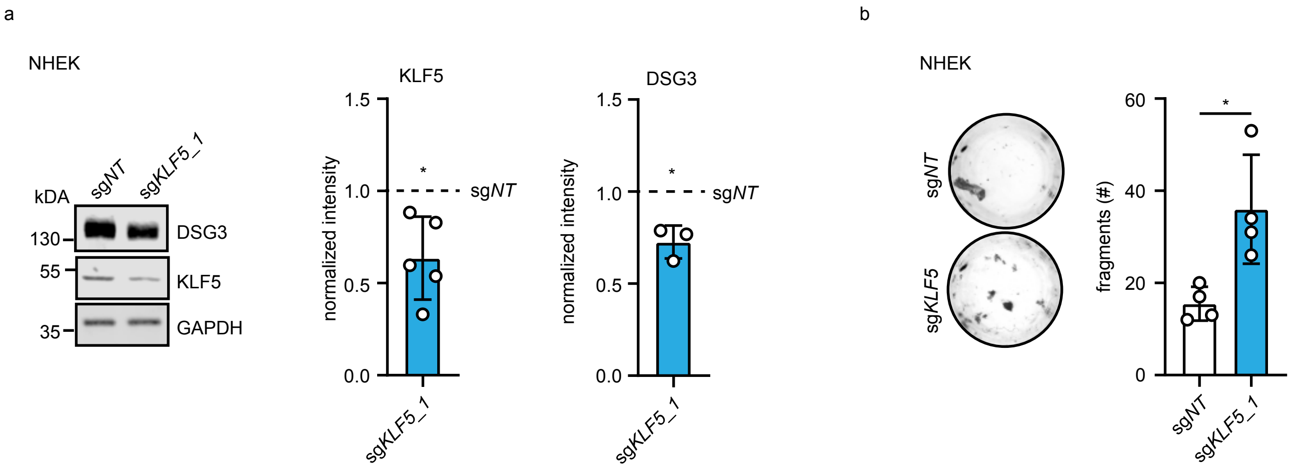

Supplemental Figure 3: a) Western blot analysis of NHEK cell lysates using KLF5, DSG3 and GAPDH antibodies. NHEKs were transduced with sg*NT* or sg*KLF5_1*. Representative Western blot images and quantifications of respective proteins are shown (KLF5 n=5 p=0.0224, DSG3 n=3 p=0.0341). b) Dispase-based dissociation assays of NHEK cells stably transduced with sg*NT* or sg*KLF5_1* plasmids (n=4, p=0.0161). Representative images and number of fragments are shown. Values expressed as mean with standard deviation (mean+/-SD). One n represents one biological replicate. Source data are provided as a Source Data file. Experiment a) was analyzed with two-sided one sample t-test, b) was analyzed with student`s t-test. $p<0.05$ *

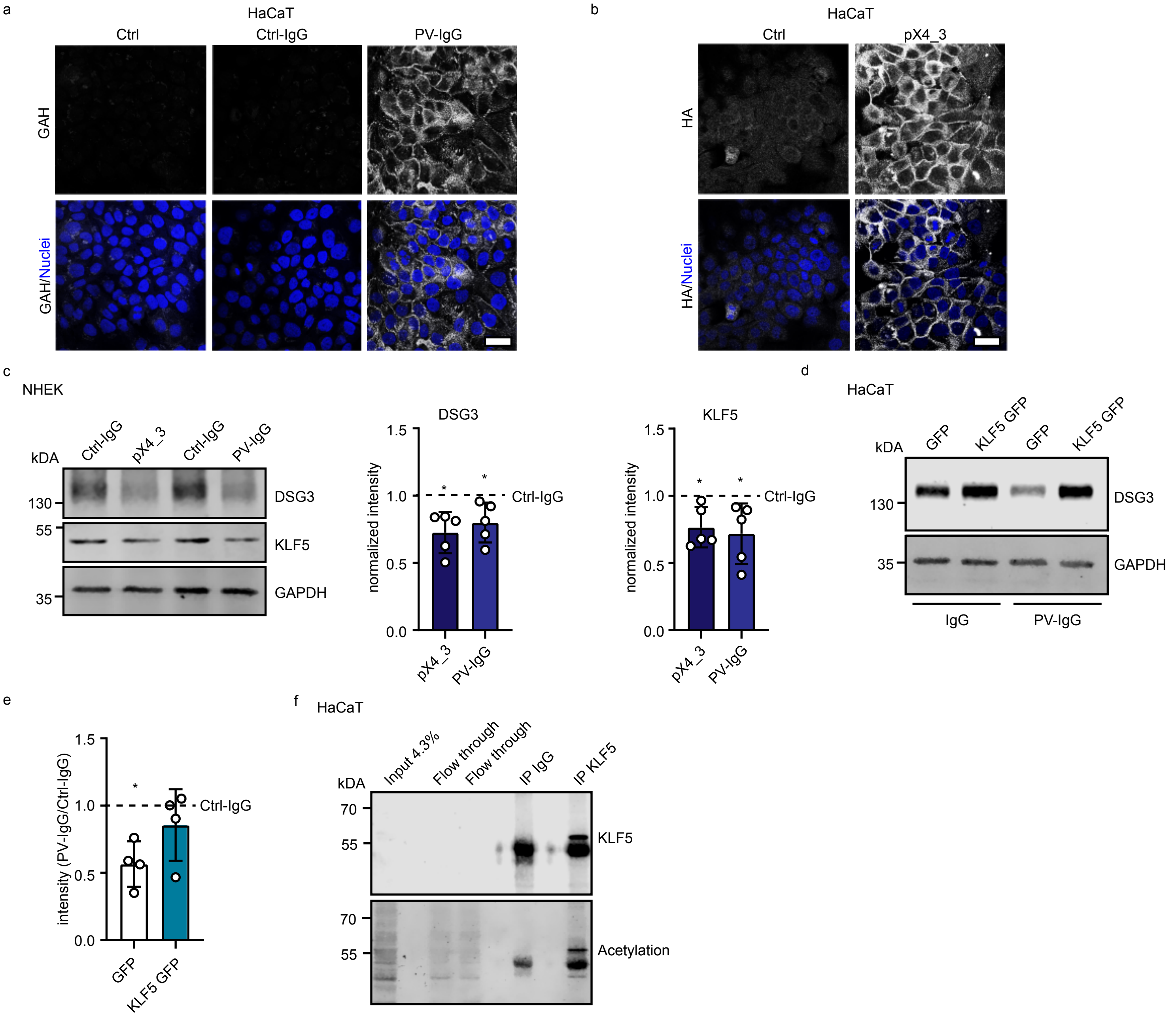

Supplemental Figure 4: a) Immunofluorescence staining of HaCaT cells incubated with Ctrl-IgG or PV-IgG for 24h. GAH-Alexa 647 antibodies served to detect bound antibodies, DAPI to visualize nuclei. Scale bar = 10 µm. b) Immunofluorescence staining of HaCaT cells incubated with Ctrl-IgG or pX4_3. GAH-Alexa647 and a HA antibody (secondary antibody Goat-anti-mouse 568) were used to detect Ctrl-IgG and pX4_3, respectively. DAPI served to visualize nuclei. Scale bar = 10 µm. c) Western blot analysis of NHEK cell lysates using KLF5, DSG3 and GAPDH antibodies. NHEKs were treated with Ctrl-IgG, pX4_3 or PV-IgG. Representative Western blot images and quantifications of respective proteins are shown (n=5, DSG3 pX4_3 p=0.0159, DSG3 PV-IgG p=0.0396, KLF5 pX4_3 p=0.0256, KLF5 PV-IgG p=0.0492). d) Western blot analysis of HaCaT cells stably transduced with KLF5-GFP or GFP as control and treated with IgG or PV-IgG. Antibodies against DSG3 or GAPDH were used (n=4). e) Quantification of Western blot shown in d) (n=4, GFP p=0.0141). f) Immunoprecipitation analysis to identify KLF5 acetylation. KLF5 was immunoprecipitated using KLF5 antibodies and the acetylation was visualized using a pan-acetylation antibody. Immunoprecipitation with Ctrl-IgGs served as control. Values expressed as mean with standard deviation (mean+/-SD). One n represents one biological replicate. Source data are provided as a Source Data file. Experiments c), e) were analyzed with two-sided one sample t-test. $p<0.05$ *

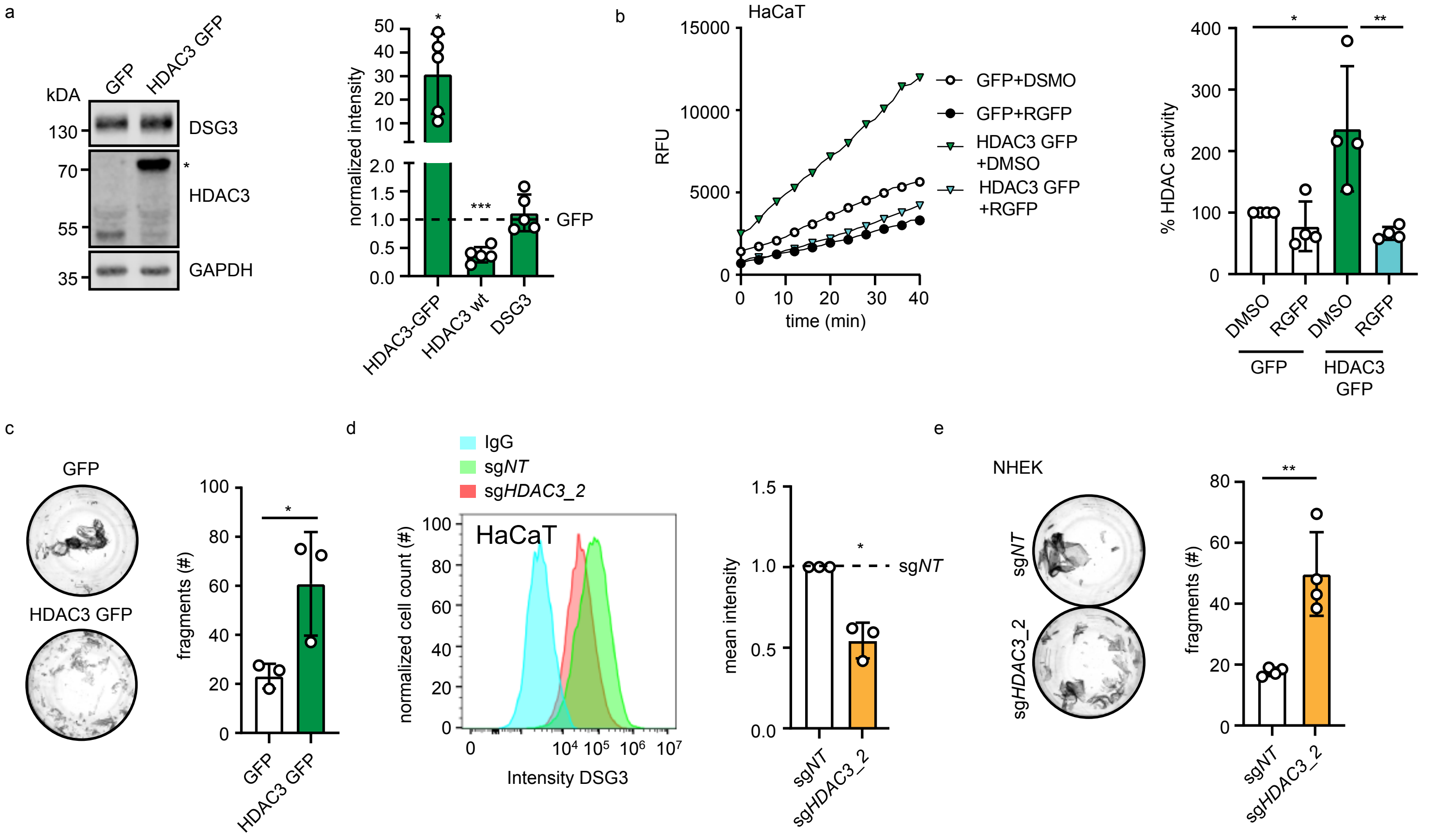

Supplemental Figure 5: a) Western blot analysis of HaCaT cell lysates stably expressing HDAC3-GFP using HDAC3, DSG3 and GAPDH antibodies. Representative Western blot images and quantifications of respective proteins (n=5, HDAC3-GFP p=0.0170, HDAC3 wt p=0.0005) are shown. Values were normalized to Ctrl. Asterix marks HDAC3-GFP. b) HDAC activity assay with HaCaT cell lysates. HaCaT cells were stably transduced with HDAC3 overexpression plasmid and treated with DMSO or 5 µM RGFP966. Two different presentations are shown. The left panel shows the increase in deacetylated peptide detected by its fluorophore over time of one representative experiment and the right panel shows the calculated HDAC activity (n=4, GFP DMSO vs HDAC3-GFP p=0.0129, HDAC3-GFP DMSO vs HDAC3 GFP RGFP966 p=0.0027). RFU relative fluorescence unit. c) Dispase-based dissociation assay of HaCaT cells stably transduced with HDAC3-GFP (n=3, p=0.0398). Representative images and number of fragments are shown. d) Flow cytometry analysis of HaCaT cells stained with DSG3 antibodies or IgG as control. HaCaT cells were stably transduced with sg*NT* or sg*HDAC3* plasmids. One representative histogram and quantification (n=3, p=0.0191) are shown. e) Dispase-based dissociation assays of NHEK cells stably transduced with sg*NT* or sg*HDAC3_2* plasmids (n=4, p=0.0037). Representative images and number of fragments are shown. Values expressed as mean with standard deviation (mean+/-SD). One n represents one biological replicate. Source data are provided as a Source Data file. Experiments a) and d) were analyzed with two-sided one sample t-test, c) and e) were analyzed with two-sided Student`s t-test, b) was analyzed with One-way-ANOVA, SIDAK correction. $p<0.05$ *; $p<0.01$ **

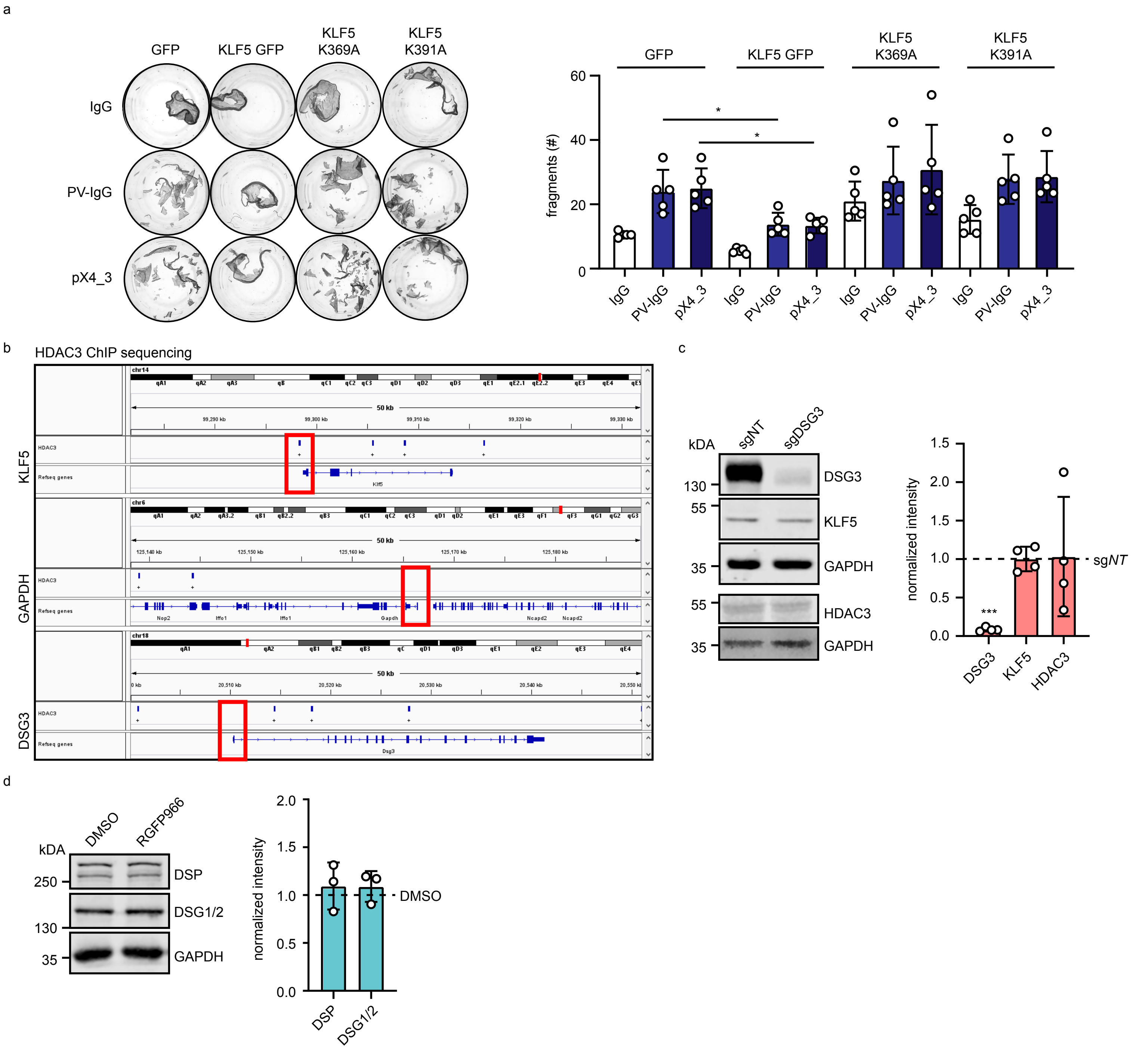

Supplemental Figure 6: a) Dispase-based dissociation assays of HaCaT cells stably transfected with KLF5, KLF5 K369A, KLF5 K391A sgKLF5_1 or GFP as control and treated with Ctrl-IgG, PV-IgG or pX4_3. Representative images and quantifications of n=5 (GFP PVIgG vs KLF5 GFP PV-IgG p=0.0255, GFP pX4_3 vs KLF5 GFP pX4_3 p=0.0117) are shown. b) HDAC3 ChIP-sequencing of mouse epidermis E18.5 dataset number (GEO number: GSE137232), KLF5, GAPDH, DSG3 gene loci are shown. c) Western blot analysis of HaCaT cells stably transduced with sg*NT* or sg*DSG3*. Antibodies against DSG3, KLF5, HDAC3 and GAPDH were used. Values were normalized to sg*NT* (n=4, DSG3 p<0.0001). d) Western blot analysis of HaCaT cells treated with DMSO or 5 µM RGFP966. Antibodies against DSP, DSG1/2 and GAPDH were used. Values were normalized to DSMO (n=4). Values expressed as mean with standard deviation (mean+/-SD). One n represents one biological replicate. Source data are provided as a Source Data file. Experiment a) was analyzed with One-way-ANOVA, SIDAK correction. c) was analyzed with one sample t-test. p<0.05 *; p<0.001 ***

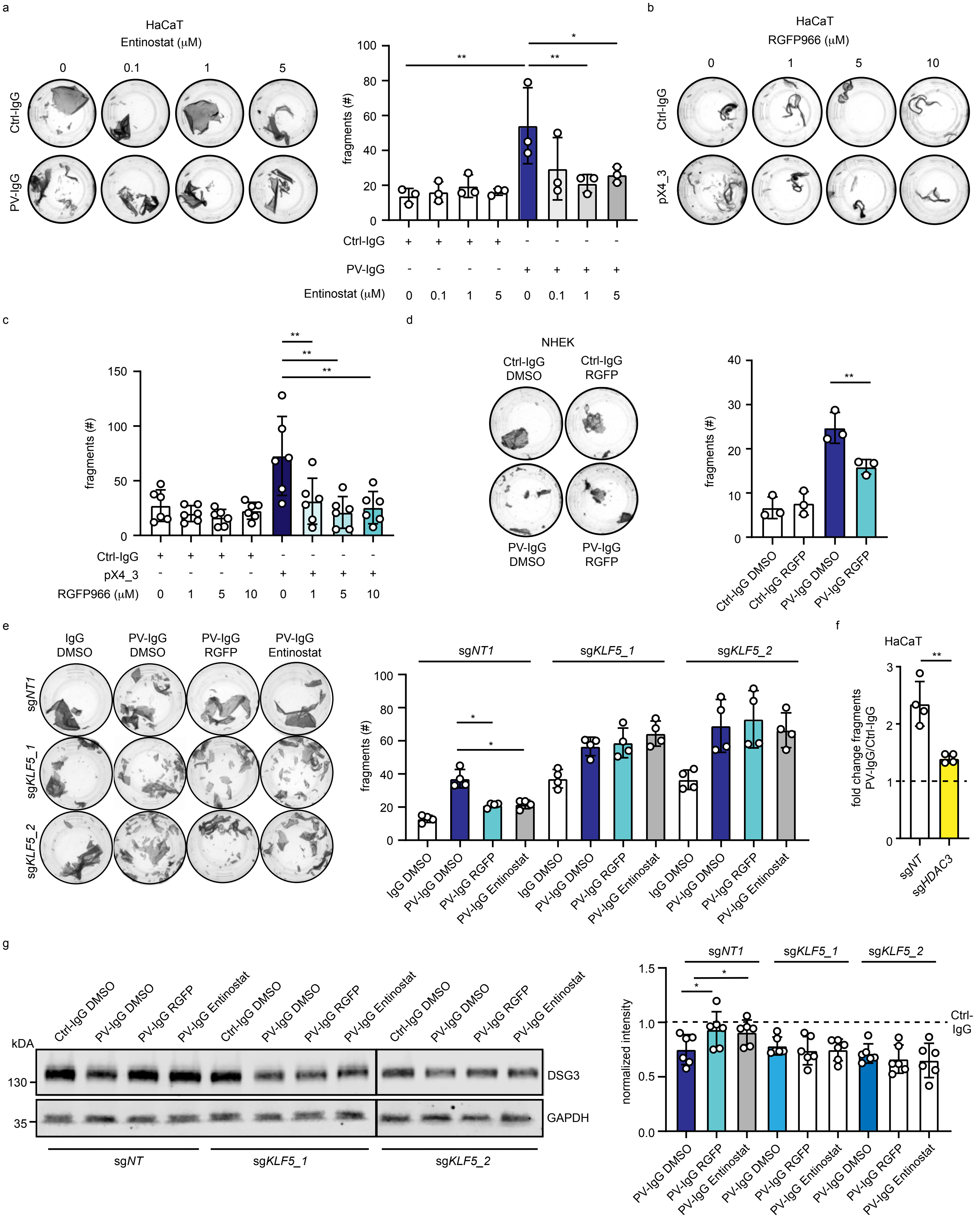

a
HaCaT
Entinostat (μM)
0
0.1
1
5
Ctrl-IgG
PV-IgG
fragments (#)
Ctrl-IgG
PV-IgG
Entinostat (μM)
b
HaCaT
RGFP966 (μM)
0
1
5
10
Ctrl-IgG
pX4_3
c
fragments (#)
Ctrl-IgG
pX4_3
RGFP966 (μM)
d
NHEK
Ctrl-IgG DMSO
Ctrl-IgG RGFP
PV-IgG DMSO
PV-IgG RGFP
fragments (#)
e
IgG DMSO
PV-IgG DMSO
PV-IgG RGFP
PV-IgG Entinostat
sgNT1
sgKLF5_1
sgKLF5_2
fragments (#)
f
HaCaT
fold change fragments PV-IgG/Ctrl-IgG
sgNT
sgHDAC3
g
Ctrl-IgG DMSO
PV-IgG DMSO
PV-IgG RGFP
PV-IgG Entinostat
kDA
130
35
DSG3
GAPDH
sgNT
sgKLF5_1
sgKLF5_2
normalized intensity
Ctrl-IgG

Supplemental Figure 7: a) Dispase-based dissociation assays of HaCaT cells treated with Ctrl-IgG or PV-IgG and indicated concentrations of the HDAC1/3 inhibitor Entinostat. Representative images and quantifications of (n=3, Ctrl-IgG vs PV-IgG p=0.013, PV-IgG vs PV-IgG 1 μM Entinostat p=0.0070, PV-IgG vs PV-IgG 5 μM Entinostat p=0.229) are shown. b) Dispase-based dissociation assays of HaCaT cells treated with Ctrl-IgG or pX4_3 and indicated concentrations of HDAC3 inhibitor RGFP966. Representative images and quantifications c) of n=6 (pX4_3 vs pX4_3 1 μM RGFP966 p=0.098, pX4_3 vs pX4_3 5 μM RGFP966 p=0.0027, pX4_3 vs pX4_3 10 μM RGFP966 p=0.0054) are shown. d) Dispase-based dissociation assays of NHEK cells treated with Ctrl-IgG or PV-IgG and 5 μM RGFP966 (n=3, PV-IgG DMSO vs PV-IgG RGFP966 p=0.0035). Representative images and number of fragments are shown. e) Dispase-based dissociation assays of HaCaT cells stably transfected with sg*NT1*, sg*KLF5_1* or sg*KLF5_2* and treated with Ctrl-IgG or PV-IgG and DMSO, 5 μM RGFP966 or 10 μM Entinostat. Representative images and quantifications of (n=4, sg*NT* PV-IgG DMSO vs sg*NT* PV-IgG RGFP966 p=0.0301, sg*NT* PV-IgG DMSO vs sg*NT* PV-IgG Entinostat p=0.0364) are shown. f) Dispase-based dissociation assays of HaCaT cells stably transfected with sg*NT* or sg*HDAC3_1* plasmid. The cells were treated for 24h with Ctrl-IgG or PV-IgG. Fold change of fragments vs. Ctrl-IgG is shown (n=4, p=0.0030). g) Western blot analysis of HaCaT cells stably transfected with sg*NT1*, sg*KLF5_1* or sg*KLF5_2* and treated with Ctrl-IgG or PV-IgG and DMSO, 5 μM RGFP966 or 10 μM Entinostat using antibodies against DSG3 and GAPDH. Representative images and quantifications are shown (n=6, sg*NT* PV-IgG DMSO vs sg*NT* PV-IgG RGFP966 p=0.0154, sg*NT* PV-IgG DMSO vs sg*NT* PV-IgG Entinostat p=0.0353). Values expressed as mean with standard deviation (mean+/-SD). One n represents one biological replicate. Source data are provided as a Source Data file. Experiments a-e) and g) were analyzed with One-way-ANOVA, SIDAK correction. f) was analyzed with two-sided Student`s t-test, p<0.05 *; p<0.01 **

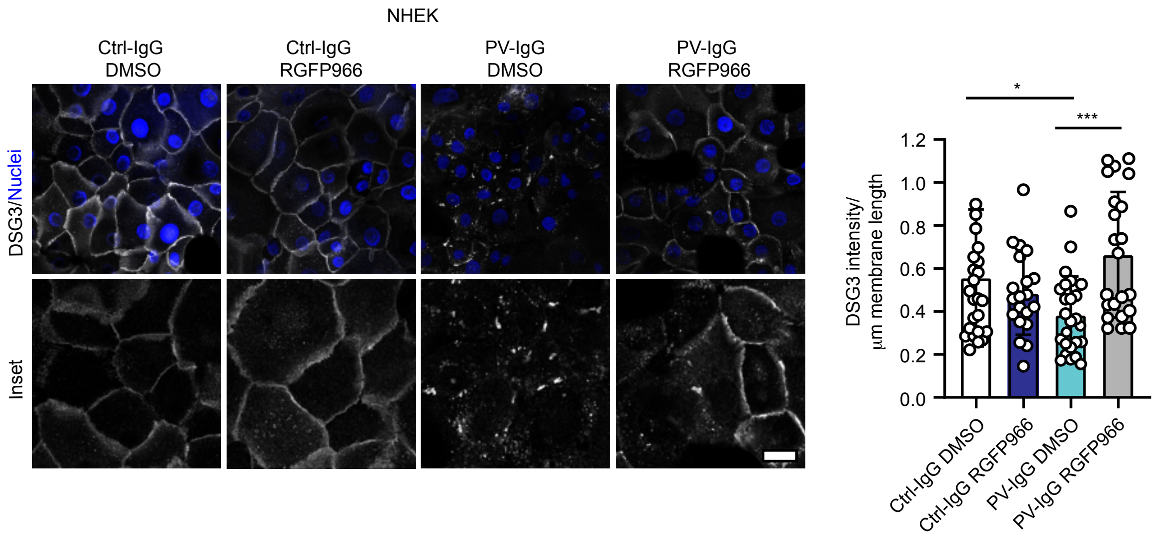

Supplemental Figure 8: Immunofluorescence staining of NHEK cells treated with Ctrl-IgG or PV-IgG and DMSO, 5 µM RGFP966 or 10 µM Entinostat using DSG3 antibodies and DAPI. Scale bar = 10 µm. Quantification of DSG3 intensity/membrane length of n=3 (Ctrl-IgG DMSO vs Ctrl-IgG RGFP966 p=0.0491, PV-IgG DMSO vs PV-IgG RGFP966 p=0.0008) is shown. Values expressed as mean with standard deviation (mean+/-SD). One n represents one biological replicate. Source data are provided as a Source Data file. One-way-ANOVA, SIDAK correction. p<0.05 *; p<0.001 ***

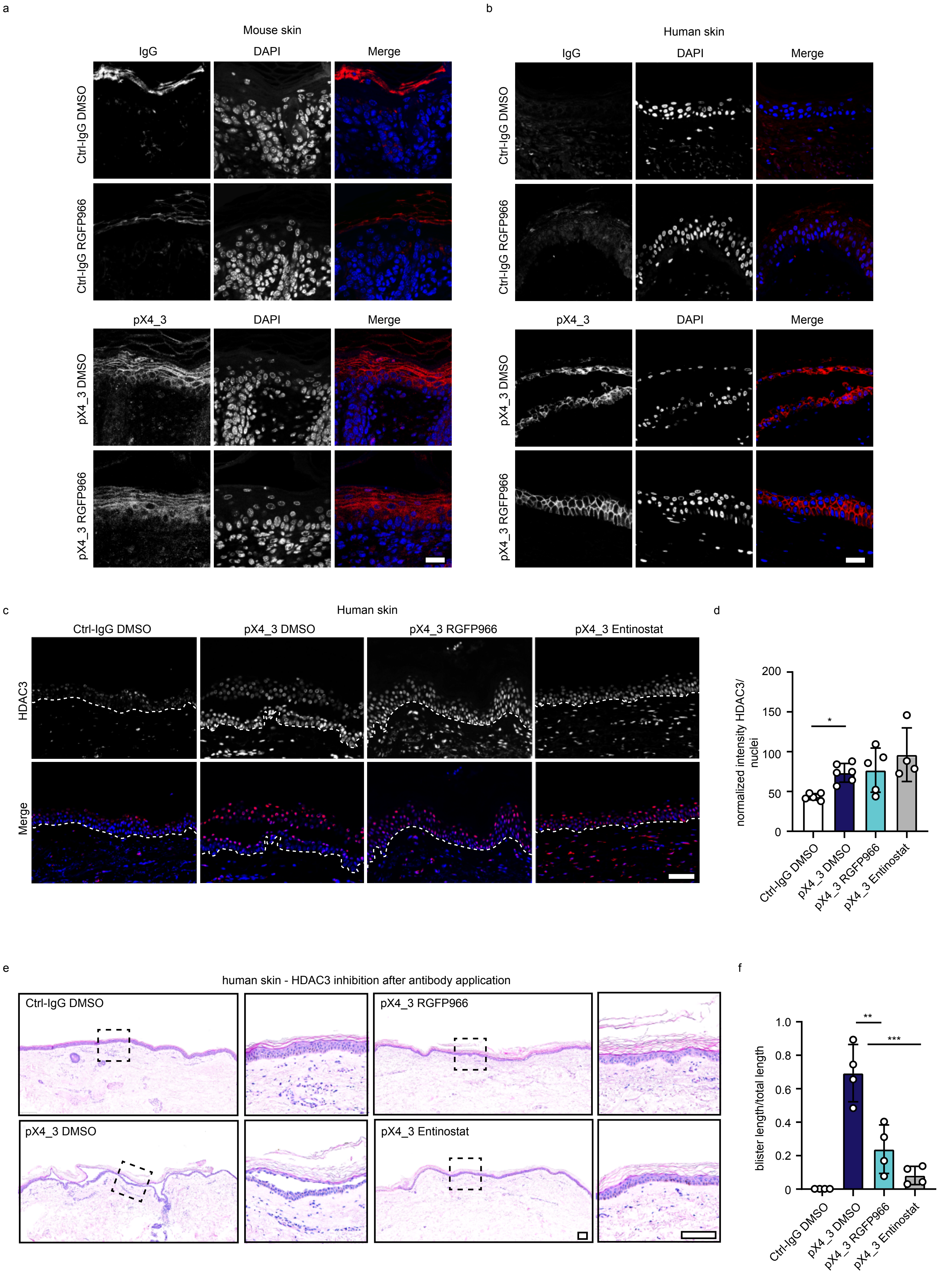
a
Mouse skin
IgG
DAPI
Merge
Ctrl-IgG DMSO
Ctrl-IgG RGFP966
pX4_3
pX4_3 DMSO
pX4_3 RGFP966
b
Human skin
c
Human skin
Ctrl-IgG DMSO
pX4_3 DMSO
pX4_3 RGFP966
pX4_3 Entinostat
HDAC3
Merge
d
normalized intensity HDAC3/nuclei
200
150
100
50
0
*
e
human skin - HDAC3 inhibition after antibody application
f
blister length/total length
1.0
0.8
0.6
0.4
0.2
0
**
***

Supplemental Figure 9: a) Immunofluorescence staining of mouse skin sections incubated with Ctrl-IgG or pX4_3 and DSMO or 5 μM RGFP966. GAH-Alexa647 was used to detect Ctrl-IgG, HA antibodies (secondary antibody Goat-anti-mouse 568) were applied to visualize bound pX4_3. DAPI served to stain nuclei. Scale bar = 100 μm. b) Immunofluorescence staining of human skin sections treated with Ctrl-IgG or pX4_3 and DSMO or 5 μM RGFP966. GAH-Alexa647 was used to detect Ctrl-IgG, HA antibodies (secondary antibody Goat-anti-mouse 568) were applied to visualize bound pX4_3. DAPI served to stain nuclei. Representative images are shown. Scale bar = 100 μm. c) Immunofluorescence staining of human skin sections incubated with Ctrl-IgG or pX4_3 and DSMO or 5 μM RGFP966. HDAC3 antibodies (secondary antibody Goat-anti-mouse 568) were applied. DAPI served to stain nuclei. Scale bar = 100 μm d) Quantification of c) displaying the normalized HDAC3 intensity per nucleus (Ctrl-IgG DMSO n=5, pX4_3 DMSO n=6, pX4_3 RGFP966 n=5, pX4_3 Entinostat n=4, Ctrl-IgG vs pX4_3 DMSO p=0.0431). e) Ex-vivo skin model: H&E staining of human skin treated with Ctrl-IgG or pX4_3 and after 2h of incubation at 37°C treated with DMSO/ 5 μM RGFP966 or 10 μM Entinostat is displayed. Scale bar = 100 μm f) Quantification of blister length versus total section length is shown (n=4, pX4_3 DMSO vs pX4_3 RGFP966 p=0.0019, pX4_3 DMSO vs pX4_3 Entinostat p=0.002). Values expressed as mean with standard deviation (mean+/-SD). One n represents one biological replicate. Source data are provided as a Source Data file. Experiments d) and f) were analyzed with One-way-ANOVA, SIDAK correction. $p<0.05$ *; $p<0.01$ **; $p<0.001$ ***

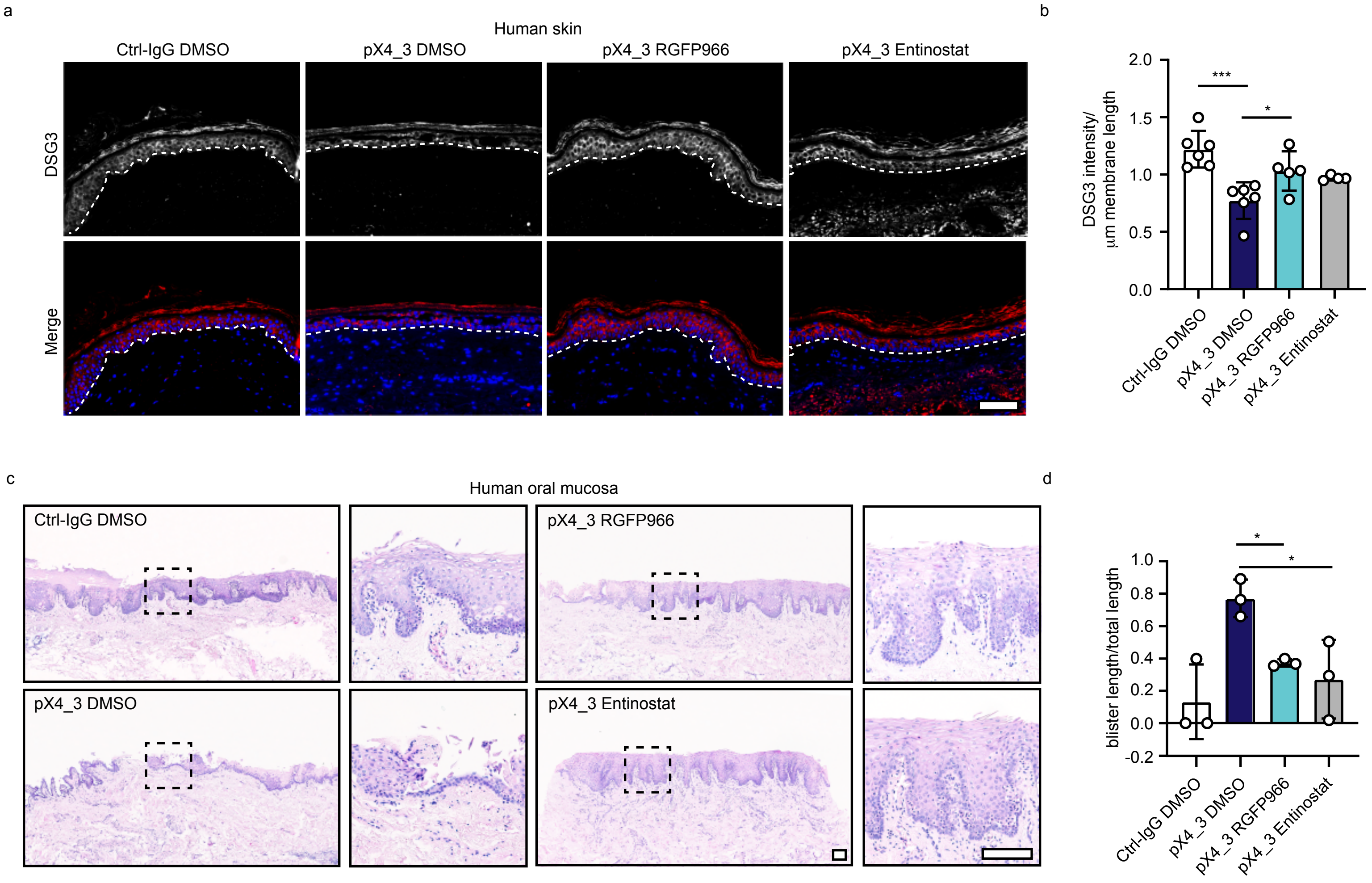

Supplemental Figure 10: a) Immunofluorescence staining of human skin sections incubated with Ctrl-IgG or pX4_3 and DSMO or 5 µM RGFP966. DSG3 antibodies (secondary antibody Goat-anti-mouse 568) were applied. DAPI served to stain nuclei. Scale bar = 100 µm b) Quantification of a) displaying the DSG3 intensity per µm membrane length (Ctrl-IgG n=6, pX4_3 DMSO n=6, pX4_3 RGFP966 n=6, pX4_3 Entinostat n=4, Ctrl-IgG DMSO vs pX4_3 DMSO p=0.0002, pX4_3 DMSO vs pX4_3 RGFP966 p=0.0314). c) Ex-vivo mucosa model: H&E staining of human oral mucosa treated with Ctrl-IgG or pX4_3 and DMSO/ 5 µM RGFP966 or 10 µM Entinostat is displayed. Scale bar = 100 µm. d) Quantification of blister length versus total section length is shown (n=3, pX4_3 DMSO vs pX4_3 RGFP966 p=0.0496, pX4_3 DMSO vs pX4_3 Entinostat p=0.0175). Source data are provided as a Source Data file. Experiments b) and d) were analyzed with One-way-ANOVA, SIDAK correction. p<0.05 *; p<0.01 **

id	neg_fc	pos_fdr
ARPC4	3.9553	0.00165
AP2S1	5.5102	0.00165
DSG3	5.2166	0.00165
NF2	3.9448	0.003713
EGFR	5.4117	0.00495
CMTR1	4.6481	0.007426
AP3B1	3.8373	0.011757
KLF5	4.3182	0.011757
RNF146	2.7636	0.012651
SHOC2	2.871	0.053694
ATP2C1	2.7762	0.053694
RHBDL1	2.8959	0.053694
SYNCRIP	2.7847	0.053694
ACACA	3.0057	0.066832
FAM103A1	5.1385	0.068379
C18orf8	2.1805	0.068379
RBPJ	2.9654	0.078334
PDPK1	4.4807	0.078658
EIF3L	3.8125	0.082583
BUD13	3.9491	0.082583
GOPC	3.0091	0.082583
HCFC1	3.0327	0.082583
OR51E2	-0.99106	0.088033
GRB2	3.5158	0.093853
PSENEN	2.5645	0.09604
RALGAPB	2.0945	0.10066
JUP	1.74	0.10066
UHRF1	3.2828	0.102016
THAP1	4.7987	0.106692
ATP5B	3.2855	0.114026
PRRT2	2.3693	0.123762
YARS	4.0844	0.14836
REV1	3.7827	0.155266
LRP3	3.5426	0.158014
ASNA1	2.4176	0.158014
ADAR	1.8122	0.158014
DYNLL1	2.4019	0.158014
AP2A1	3.6686	0.164018
EIF3E	2.9876	0.167936
DDX51	2.1814	0.173267
HDAC3	3.0909	0.173267
AMD1	3.7205	0.173267
OSBPL9	3.0723	0.173267
TCEB2	2.1425	0.173267
PTPN11	3.6671	0.173267
VPS35	2.111	0.173267

ELANE	0.4664	0.173267
TAF6L	1.4917	0.184455
SPCS1	2.8643	0.184455
AP3D1	2.6205	0.184455
MSL1	3.1478	0.193511
NFATC2IP	2.1921	0.193511
UBE2K	3.2026	0.193511
FCHO2	2.4606	0.193511
ABT1	2.5589	0.193511
ZNF286A	3.1434	0.193511

Supplementary Table 1: Putative positive regulators of DSG3 identified by CRISPR/Cas9 sgRNA library screening. Gene name, fold change and false discovery rate calculated using MAGeCK are displayed.

Gene.names	difference_Dsg3_control	pvalue_Dsg3_control
RECQL	2.413126226	1.01E-08
HNRNPU	2.27298905	8.36E-08
SNRPA	2.957743483	2.35E-07
RPA2	3.328007594	1.84E-06
KLF5	3.867606607	2.23E-06
C5orf24	2.610612423	2.36E-06
HNRNPDL	4.411822249	2.40E-06
PNKP	1.250201498	1.79E-05
RBM14	4.563278673	1.84E-05
PURA	4.273307523	1.92E-05
TFG	4.345233953	2.02E-05
SSBP1	1.578867556	2.30E-05
TAF15	4.357941564	2.55E-05
TOP3A	2.780121566	2.63E-05
HNRNPA3	2.723586621	3.94E-05
LRRC59	3.001649146	5.03E-05
HNRNPM	1.289179435	5.27E-05
APOBEC3C	1.129300343	6.58E-05
HNRNPAB	1.871597676	7.57E-05
HNRNPA0	1.168710402	8.25E-05
HNRNPUL2	2.26652583	9.68E-05
HNRNPA2B1	2.058903964	0.000122612
MYL6	1.585873079	0.000134425
TREX1	2.276283354	0.000156838
RBM4;RBM4B	1.889639939	0.00017215
FAM120A	6.24815391	0.00018127
PHF10	3.121668126	0.000207259
KCTD1	4.114620268	0.000229114
HNRNPUL1	1.105832488	0.000356252
FOSL1	2.684540776	0.000478254
IGF2BP3	1.296282011	0.000528347
PRPF31	1.300414729	0.000637683
POP4	1.368762823	0.000654675
HNRNPA1;HNRNPA1L2	1.658097109	0.000660846
PCCA	2.310543037	0.000664104
ZNF362	1.363742032	0.000730627
MBNL1;MBLL;MBNL2	1.527691944	0.000731441
APOBEC3B	2.602608391	0.0007938
SCAF4	3.043807721	0.000840745
POLB	4.996980607	0.001221146
ZNF512B	1.263948847	0.001447336
EWSR1	3.947721973	0.00256576
TCF7L2	1.390263089	0.003260633
FMR1	2.658846642	0.003331362
DDX3X;DDX3Y	1.152450634	0.003348867
RUNX1	1.255463857	0.003819507
HNRNPK	1.18291548	0.003869622
TCF20	1.077084018	0.004160622

RBMX;RBMXL1	4.817836097	0.005783032
RMI1	1.424904639	0.006001566
FUS	1.517851604	0.006567697
PDS5A;PDS5B	1.335595597	0.006772364
CD2BP2	1.609465929	0.006884864
YWHAQ	1.636474797	0.00725385
RPA1	1.469317078	0.016627644
IGHMBP2	1.776833826	0.026980652
HMG20B	1.526109457	0.03239257
PPL	4.947517984	0.044159035

Supplementary Table 2: Putative binders of the DSG3 promoter identified by promoter pulldown followed by mass spectrometry, fold change and p-value are displayed. The putative binders were identified using two-sided Welch-Test.

Gene.names	difference_Dsg2_control	pvalue_Dsg2_control
HNRNPAB	2.989246736	3.86E-08
HNRNPA0	1.556165942	6.44E-07
HNRNPM	2.161430548	1.49E-06
PDCD11	1.064040942	2.78E-06
HNRNPDL	4.767523499	3.09E-06
BAP1	2.709821543	3.48E-06
SSBP1	2.652948293	3.63E-06
RBM3	4.174619278	6.32E-06
BLM	1.137069888	7.07E-06
CIRBP	3.389542958	8.59E-06
TOP3A	3.163113132	1.32E-05
REEP5	2.24094676	2.57E-05
PCCA	2.654090445	2.91E-05
TREX1	2.043100062	4.45E-05
EXOSC6	1.78876786	4.81E-05
PELP1	1.397486474	5.02E-05
LGALS1	2.267802178	6.19E-05
RPA2	4.818090291	6.40E-05
RBM19	3.566799172	7.81E-05
PURA	4.646673825	8.94E-05
C17orf49;BAP18;RNASEK-C17orf49	1.905507	9.66E-05
ERI1	1.204466955	0.000107246
POP7	2.667553561	0.000107392
PHF10	3.527344385	0.000107943
KCTD1	4.789936858	0.000157204
EXOSC7	2.544726317	0.00020839
DIDO1	1.498410537	0.000246265
RBM4;RBM4B	3.100036213	0.000246306
RBM28	1.165418869	0.000333318
SRSF7	1.436109938	0.000397243
SCAF4	3.13314622	0.000494602
SRRM2	1.693387652	0.000587667
TAF1D	1.65007595	0.000726518
WASF2	1.089830711	0.00078081
PABPC4	1.515056038	0.000814476
RPA3	3.236781334	0.000823869
CCNK	3.059320374	0.000953756
HMGXB4	1.526084981	0.001032843
RMI1	1.899180633	0.001086773
RBM14	2.414576882	0.001115679
POP4	1.678031844	0.001379555
WDR82	1.056783076	0.001412727
RPA1	3.631130988	0.001460378
POLR2H	1.434687862	0.001605603
BTF3	2.153494074	0.001971554
PURB	2.039802124	0.002185862
PRPF31	1.210461886	0.002222
TCF7L2	1.394364624	0.002352731

CLIC1	1.298860851	0.003491713
ASPH	1.568086543	0.003857263
ARF6	1.254187881	0.004100422
GMPS	1.848398481	0.005106305
PDS5A;PDS5B	1.46434627	0.005684908
CDK13	1.440739013	0.006724652
YWHAQ	1.744095347	0.009200404
RPL12	1.160119802	0.010512706
TAF8	1.539796059	0.013261826
TAF10	2.900203254	0.014028763
RNF213	2.187961561	0.023757696
PDZD2	2.559478908	0.029779191
LRRC59	2.193919801	0.036040691
RBMX;RBMXL1	2.44775552	0.037624643

Supplementary Table 3: Putative binders of the DSG2 promoter identified by promoter pulldown followed by mass spectrometry, fold change and p-value are displayed. The putative binders were identified using two-sided Welch-test.

Sample	Localisation	Category	Gender (m/f)	anti-DSG3	anti-DSG1
1	skin	Ctrl	f	na	na
2	skin	Ctrl	m	na	na
3	skin	Ctrl	f	na	na
4	skin	Ctrl	f	na	na
5	skin	Ctrl	f	na	na
6	skin	Ctrl	f	na	na
7	skin	Ctrl	m	na	na
1	skin	Pemphigus vulgaris	m	949	143
2	skin	Pemphigus vulgaris	f	93	10
3	skin	Pemphigus vulgaris	f	141	861
4	skin	Pemphigus vulgaris	f	935	737
5	skin	Pemphigus vulgaris	f	178	3
6	skin	Pemphigus vulgaris	m	118	<20
7	skin	Pemphigus vulgaris	m	118	<20
1	mucosa	Ctrl	m	na	na
2	mucosa	Ctrl	f	na	na
3	mucosa	Ctrl	f	na	na
4	mucosa	Ctrl	f	na	na
5	mucosa	Ctrl	m	na	na
1	mucosa	Pemphigus vulgaris	f	67	<20
2	mucosa	Pemphigus vulgaris	f	1893	143
3	mucosa	Pemphigus vulgaris	m	224	<20
4	mucosa	Pemphigus vulgaris	m	31	<20
5	mucosa	Pemphigus vulgaris	m	556	<20

Supplementary Table 4: Clinical and immunological profile of Ctrl and pemphigus vulgaris patients of skin and mucosa. Gender and levels of DSG1/DSG3 antibodies are displayed.

Vector	Source	Number	Contibutor	Cloning sites	Insert sequence
LentiCas9-Blast	Addgene	52962-LV	Feng Zhang		
psPAX2	Addgene	12259	Didier Trono		
pMD2.G	Addgene	12260	Didier Trono		
LentiCRISPR v2	Addgene	52961	Addgene		
pGL4.10	Promega	9PIE665	Wiebke Bechtel Walz		
pGL4.10 DSG3 promoter	Selfmade			Sac1, Hind3	AACAGCAAGCAGGTTCACCACACTGGGGAAAATGTCTTGAGTAATGAAAAGAGCACCAAGATAATCTACCTTATTAGGCAATCTAGGCTTTTTTGAAAGTTATAAATGAAAAGAAATACAGTATAAAAGATGTCATTTGAGTATTATCAGAGTAATTTTGCAACGCATTATTTATATATTTCAAATATTCAAGGCACCATATAACTGTTCAATTTTATTTCTTTTTTTTTTTTTATTCAAAGCCTACATTCATAGTTGCTTAATGGGATGTTTTTCTTCTTCATATTTTTAGTGCCTTTCAATAAAATATATCATTGCTTAACATCTACATTTTGCTTAACTTTAGATCTACCCACACATACTCAGGCTGAAAATAAATGCTCTTTTACAAGTTAGATTTTGGGGGATTTTGTACGTTTTGCTTTGCTTTTCGGTTTTCATTGTAACCATAGTCAACTAGATTCAGCCATAAGACATCAACACTGACAAAGTGTAGTCCGGACTAGCTTTTGCACTTCCACATCATGATTAATTTATTATTCATCTGGTACCTTCCTATTAAACTTTGTCTTTAGCCTGTGAACTTTTCTTTCAGCTTCCAACAAAGGATTTCATTGCTTTAGGAATCGAGGTGTGGCTAAGTTTTTACCTGGGAGCAAAGGT[illegible]
pGL4.10 DSG2 promoter	Selfmade			Sac1, Hind3	GTGCGTTGGTCGCTATTAACTCCATTTGACAGATCTGGAAACTGGGGCCCAGAGAGGCTAGTCCAGGCCACTCAGCTAGTCACTCTTGGAGCGGGGCCTCTGATCTAGCTAGTGGTGTAATAGAAATTGTAAATCATTTCAAAGAATATATATATTTCCCGTAACACCCTAGAGTTTTCTCCTAGCCCAAGAATTGTCCTTTTTGACCTCTGGTGATTGTTTCTCCTTGAGCCAGATAAGCTGCAAACCTGTTGGAAGCAGTGAAGGGCCATAGGGAAGAGCAGCCTTTGGGGAGACAGCCACCTGAAATTCTGCTCCACAACCTCCGCTCCAGGAGAGCGGGCCGAACGCACCGCGGTCGCAGCTTTCCTGAGGACACGGGTCCCCATGTCACTAGCGACACCTGCGCTCCGGGCCCAAAGCACAATCACAAAGTGCGGGACCTTTGGGCGGTGCTGGGCGGCCTGGCCCCGCAGCCACTGGGCCGGTTGGTCCCGCAGGACTCTGCTCCGTGGAGCTTTTGTCCTTGGGCCGGGGCCCTGCGACCCGCGCTACCCAGGTCGAGCCCCGCCAGGAGGCTCCCTCCTCCCGCCATTCTCCAGAGAGTAGGGGGTCGGGAAGGAGAGAACGGCGGGGCGGGGCCTGCCACCCACAGTCCCCAACCCAAGGACGTCACGGTCCCAGGTGCGAGTGCGAGCGGGCGGGAGTCCACCCCGCGCCTCCCTCCTCTGCTGAACTCCCCACCTCTG[illegible]
pGL4.73	Promega	9PIE691	Wiebke Bechtel Walz		
LentiCRISPR v2 sgNT	Selfmade			Esp3I	CTGAAAAAGAAGGAGTTGA
LentiCRISPR v2 sgKLF5_1	Selfmade			Esp3I	GCTGTCTGATTTGTAGAACT
LentiCRISPR v2 sgKLF5_2	Selfmade			Esp3I	GTCTTGATGTGTGTTACGCA
LentiCRISPR v2 sgHDAC3_1	Selfmade			Esp3I	TGGGTCAATGCCAGGCGATG
LentiCRISPR v2 sgHDAC3_2	Selfmade			Esp3I	ACCTGGAGCACAATGCACGT
LentiCRISPR v2 sgDSG3	Selfmade			Esp3I	AGAGAAACCACTTATACTAA
LentiCRISPR v2 sgRAPGEF1	Selfmade			Esp3I	ATGTTGACTGTTACGCACAG
LentiCRISPR v2 sgADAR	Selfmade			Esp3I	GTGCATACACTCAAGCAGTG
LentiCRISPR v2 sgAP2S1	Selfmade			Esp3I	GGCCATTCACAACTTCGTGG
LentiCRISPR v2 sgAP3B1	Selfmade			Esp3I	ACATGCTAACTCGATATGCT
LentiCRISPR v2 sgEGFR	Selfmade			Esp3I	GTCTGCGTACTTCCAGACCA
LentiCRISPR v2 sgPDPK1	Selfmade			Esp3I	GTTCTTCGAGTCCGTCACGT
LentiCRISPR v2 sgRBPJ	Selfmade			Esp3I	CATTGCCTCAGGAACAAAGG
pLenti_C_mGFP	OriGene Technologies	PS100071			
pLenti_HDAC3_C_mGFP	Selfmade			Selfmade	ORF NM_003883 w/o STOP
pLenti_KLF5_C_mGFP	Selfmade			Selfmade	ORF NM_001730 w/o STOP
EF1a-hKLF5-P2A-HygR-Barcode	Addgene	120491			
pKLV2-U6gRNA5(gGFP)-PGKBFP2AGFP-W	Addgene	67980	Kosuke Yusa		
LentiGuide-Puro	Addgene	73178-LV	David Root and John Doench		

Target	Purpose	Sequence forward	Sequence reverse
GAPDH	qRT-PCR	GACAACAGCCTCAAGATCATCAG	GAGTCCTTCCACGATACCAAAGT
KLF5 1	qRT-PCR	GAATCCGGCCCGCGAC	CTGGAGGAAGCTGAGGTGTC
KLF5 2	qRT-PCR	CCGCGAATCCGGCCC	TGGAGGAAGCTGAGGTGTCA
KLF5 3	qRT-PCR	AATCCGGCCCGCGAC	GGAACTGGAGGAAGCTGAGG
KLF5 4	qRT-PCR	GCGAATCCGGCCCGC	ACTGGAGGAAGCTGAGGTGT
HDAC3	qRT-PCR	AGTCAGCCCCACCAATATGC	TGTGTAACGCGAGCAGAACT
DSG3	qRT-PCR	CCCAGTTCCTGATGGCTCAGA	AAATCGGCTCCATTGGCTGTT
DSG3 promoter 1	ChIP-qPCR	AAGGCATGAAGAACTCACATTGC	TGCATGGGTCACACGGAAATA
DSG3 promoter 2	ChIP-qPCR	TCCGGACTAGCTTTTGCACT	GCTTGCATCACCTTTGCTCC
DSG3 promoter 3	ChIP-qPCR	TAACCATCTAACGAGGGCCAC	GCTACTGTTATGCCACCAAAAAG
DSG3 promoter 4	ChIP-qPCR	TTCAAGGCACCATATAACTGTTCAA	CAGCCTGAGTATGTGTGGGTAG
DSG3 promoter 5	ChIP-qPCR	ACCTTATTAGGCAATCTAGGCTT	TGCGTTGCAAAATTACTCTGAT
KLF5 promoter	ChIP-qPCR	AAGGCATGAAGAACTCACATTGC	TGCATGGGTCACACGGAAATA
GAPDH promoter	ChIP-qPCR	CACAGTCCAGTCCTGGGAAC	TAGTAGCCGGGCCCTACTTT
CTNNB1 promoter	ChIP-qPCR	CGCGGGGACTACTTTCCAC	TTATAAGTCGCGCAGAAGCCG
DSG2 promoter	Cloning	CGCCGCGGCGCGCCGTGCGTTGGTCGCTATTAACT	GCGGCGCTCGAGGCCGCTTTTACAGAGGTGGG
DSG3 promoter	Cloning	CGCCGCGGCGCGCCAACAGCAAGCAGGTTCACCA	GCGGCGCTCGAGGCTTGCATCACCTTTGCTCC
HDAC3 ORF NM_003883	Cloning	CGCCGCGGCGCGCCATGGCCAAGACCGTGGC	GCGGCGCTCGAGAATCTCCACATCGCTTTCCTTG
Negative Ctrl Promoter pulldown	Promoter pulldown	5`Bio GGGGCGAAGAAGTTGTCCAT	TGAGAGTGCACCATAGGGGA
DSG2 promoter	Promoter pulldown	5`Bio GTGCGTTGGTCGCTATTAACT	GCCGCTTTTACAGAGGTGGG
DSG3 promoter	Promoter pulldown	5`Bio AACAGCAAGCAGGTTCACCA	GCTTGCATCACCTTTGCTCC
KLF5 ORF NM_001730	Cloning	CGCCGCGGCGCGCCATGGCTACAAGGGTGCTGAGC	TACAGCCTCGAGGTTCTGGTGCCTCTTCATATGCAGG
KLF5 K369A	Mutagenesis	CGATTTGGAGGCACGACGCATC	GGGTTACTCCTTCTATTG
KLF5 K391A	Mutagenesis	TTCTCATTTAGCAGCTCACCTGAG	GACTTGGTATAAACTTTTGTG

NGS_P5 nt0	Illumina library sequencing	AATGATACGGCGACCACCGAGATCTACACTCTTTCCCTACACGACGCTCTTCCGATCTTTGTGGAAAGGACGAAACACC*G	
NGS_P5 nt1	Illumina library sequencing	AATGATACGGCGACCACCGAGATCTACACTCTTTCCCTACACGACGCTCTTCCGATCTCTTGTGGAAAGGACGAAACACC*G	
NGS_P5 nt2	Illumina library sequencing	AATGATACGGCGACCACCGAGATCTACACTCTTTCCCTACACGACGCTCTTCCGATCTGCTTGTGGAAAGGACGAAACACC*G	
NGS_P5 nt3	Illumina library sequencing	AATGATACGGCGACCACCGAGATCTACACTCTTTCCCTACACGACGCTCTTCCGATCTAGCTTGTGGAAAGGACGAAACACC*G	
NGS_P5 nt4	Illumina library sequencing	AATGATACGGCGACCACCGAGATCTACACTCTTTCCCTACACGACGCTCTTCCGATCTCAACTTGTGGAAAGGACGAAACACC*G	
NGS_P5 nt6	Illumina library sequencing	AATGATACGGCGACCACCGAGATCTACACTCTTTCCCTACACGACGCTCTTCCGATCTTGCACCTTGTGGAAAGGACGAAACACC*G	
NGS_P5 nt7	Illumina library sequencing	AATGATACGGCGACCACCGAGATCTACACTCTTTCCCTACACGACGCTCTTCCGATCTACGCAACTTGTGGAAAGGACGAAACACC*G	
NGS_P7_N701	Illumina library sequencing	CAAGCAGAAGACGGCATACGAGATTCGCCTTAGTGACTGGAGTTCAGACGTGTGCTCTTCCGATCTTCTACTATTCTTTCCCCTGCACTG*T	
NGS_P7_N702	Illumina library sequencing	CAAGCAGAAGACGGCATACGAGATCTAGTACGGTGACTGGAGTTCAGACGTGTGCTCTTCCGATCTTCTACTATTCTTTCCCCTGCACTG*T	
NGS_P7_N703	Illumina library sequencing	CAAGCAGAAGACGGCATACGAGATTTCTGCCTGTGACTGGAGTTCAGACGTGTGCTCTTCCGATCTTCTACTATTCTTTCCCCTGCACTG*T	
NGS_P7_N704	Illumina library sequencing	CAAGCAGAAGACGGCATACGAGATGCTCAGGAGTGACTGGAGTTCAGACGTGTGCTCTTCCGATCTTCTACTATTCTTTCCCCTGCACTG*T	
NGS_P7_N705	Illumina library sequencing	CAAGCAGAAGACGGCATACGAGATAGGAGTCCGTGACTGGAGTTCAGACGTGTGCTCTTCCGATCTTCTACTATTCTTTCCCCTGCACTG*T	
NGS_P7_N706	Illumina library sequencing	CAAGCAGAAGACGGCATACGAGATCATGCCTAGTGACTGGAGTTCAGACGTGTGCTCTTCCGATCTTCTACTATTCTTTCCCCTGCACTG*T	
NGS_P7_N707	Illumina library sequencing	CAAGCAGAAGACGGCATACGAGATGTAGAGAGGTGACTGGAGTTCAGACGTGTGCTCTTCCGATCTTCTACTATTCTTTCCCCTGCACTG*T	
NGS_P7_N710	Illumina library sequencing	CAAGCAGAAGACGGCATACGAGATCAGCCTCGGTGACTGGAGTTCAGACGTGTGCTCTTCCGATCTTCTACTATTCTTTCCCCTGCACTG*T	
NGS_P7_N711	Illumina library sequencing	CAAGCAGAAGACGGCATACGAGATTGCCTCTTGTGACTGGAGTTCAGACGTGTGCTCTTCCGATCTTCTACTATTCTTTCCCCTGCACTG*T	
NGS_P7_N712	Illumina library sequencing	CAAGCAGAAGACGGCATACGAGATTCCTCTACGTGACTGGAGTTCAGACGTGTGCTCTTCCGATCTTCTACTATTCTTTCCCCTGCACTG*T	
NGS_P7_N714	Illumina library sequencing	CAAGCAGAAGACGGCATACGAGATTCATGAGCGTGACTGGAGTTCAGACGTGTGCTCTTCCGATCTTCTACTATTCTTTCCCCTGCACTG*T	
NGS_P7_N715	Illumina library sequencing	CAAGCAGAAGACGGCATACGAGATCCTGAGATGTGACTGGAGTTCAGACGTGTGCTCTTCCGATCTTCTACTATTCTTTCCCCTGCACTG*T	

Supplementary Table 5: List of used plasmids with source, number and contributor and primer sequences used for qRT-PCR, ChIP-qPCR, cloning, promoter pulldown, mutagenesis and library sequencing.

Figure	Supplement	Finding
1a-b		CRISPR/Cas9 screen suggests KLF5 and 55 other proteins as positive regulators of DSG3 in the membrane.
	Supp 1a-g	Validation of CRISPR/Cas9 screen-identified candidates.
1c-d		Pulldown of the DSG3 promoter followed by quantitative mass spectrometry revealed proteins that bind directly to the DSG3 promoter.
1e		Overlap of both screens identified KLF5 as a candidate.
	Supp 2a-b	Pulldown of the DSG2 promoter followed by quantitative mass spectrometry did not identify KLF5.
	Supp 2c-d	KLF5 binds to the DSG3 promoter.
2a-e		Validation of KLF5 as regulator of DSG3 and adhesion in HaCaT cells.
	Supp 3a-b	Validation of KLF5 as regulator of DSG3 and adhesion in NHEK cells.
2f-g		Overexpression of KLF5 increases DSG3 levels and adhesion
3a-b		KLF5 levels are reduced in PV patient skin.
	Supp 4a-b	PV-IgG and pX4_3 bind to the cell membrane in HaCaT cells.
3c		PV-IgG treatment reduces KLF5 and DSG3 protein levels in HaCaTs and NHEKs.
3d	Supp 4d-e	Overexpression of KLF5 prevents PV-IgG-induced DSG3 reduction.
3e-f		PV-IgG treatment increases HDAC3 protein levels and HDAC3 activity.
3g-h		HDAC3 levels are increased in PV patients in skin.
4a	Supp 4f	KLF5 is acetylated in HaCaT cells, but PV-IgG treatment did not alter KLF5 acetylation levels.
	Supp 5a-b	HDAC3 overexpression increases HDAC3 activity and reduces cell-cell adhesion.
	Supp 5d-e	But also HDAC3 depletion reduces DSG3 protein levels and also cell-cell adhesion. Therefore a correct HDAC3 balance is needed for normal cohesion.
	Supp 6a	KLF5 lysine mutants do not rescue the PV-IgG-induced loss of cell-cell adhesion.
4b		PV-IgG treatment reduces KLF5 mRNA levels.
4d-e		PV-IgG did not induce KLF5 reduction in HDAC3 depleted cells.
4f-g		HDAC3 binds to the KLF5 promoter, which is increased by PV-IgG.
	Supp 6b	HDAC3 binds to the KLF5 promoter in a published data set.
4h		p38MAPK is regulating HDAC3 levels.
5a-b, d		Titrating the inhibitor of HDAC3, RGFP966, in HaCaT cells incubated with PV-IgG restored cell adhesion, DSG3 levels and DSG3 localization at the membrane.
5c		RGFP966 increases DSG3 promoter activity in PV-IgG-treated HaCaT cells.
	Supp 6c-d	DSG3 depletion does not influence HDAC3 or KLF5 levels. HDAC inhibition does not alter DSG1/2, and DSP content.
	Supp 7a-d	Titrating the HDAC1/3 inhibitor of HDAC3, Entinostat, or the HDAC3 inhibitor, RGFP966, to HaCaT cells incubated with PV-IgG or pX4_3, also restored cell adhesion. RGFP966 also restored cell-cell-adhesion in NHEK cells.
	Supp 7e	Depletion of KLF5 prevented prevented the protective effevt of the HDAC3 inhibitors RGFP966 or Entinostat in PV-IgG-treated HaCaT cells.
	Supp 7f	Depletion of HDAC3 prevented the PV-IgG-mediated loss of cell-cell adhesion.
	Supp 7g	Depletion of KLF5 prevented rescue of DSG3 protein levels in PV-IgG HaCaT cells by HDAC3 Inhibitors RGFP966 or Entinostat.
	Supp 8	Titrating the inhibitor of HDAC3, RGFP966, to HaCaT cells incubated with PV-IgG , restored DSG3 levels at the membrane in NHEK cells.
6a-d		RGFP966 or Entinostat treatment prevented pX4_3 induced blistering in a neonatal PV mouse model and in human ex-vivo PV skin models.
	Supp 9a-b	pX4_3 binds to the membrane of keratinocytes in human and mouse skin.
	Supp 9c-d	pX4_3 induces HDAC3 increase in the human ex-vivo skin model.
	Supp 9e	Injection of RGFP966 inhibitor two h after pX4_3 injection also prevents blistering in the ex-vivo human skin model.
6e		pX4_3 reduces KLF5 levels in the ex vivo human skin model, which is prevented by the HDAC3 inhibitors RGFP966 or Entinostat.
	Supp 10a-b	pX4_3 reduces DSG3 levels in the ex vivo human skin model, which is ameliorated by the HDAC3 inhibitors RGFP966 or Entinostat.
	Supp 10c-d	Treatment with RGFP966 or Entinostat prevented pX4_3-induced blister formation in human ex vivo mucosal models.
6g		Graphical summary

Supplementary Table 6: Summary of results for all Figures and Supplementary Figures.
