# Supplementary material for: Unbiased screening identifies regulators of cell-cell adhesion and treatment options in pemphigus
Source: Nat Commun. 2024 Sep 14;15:8044. doi: 10.1038/s41467-024-51747-2 (PMC11399147; doi:10.1038/s41467-024-51747-2)
Supplement: Supplementary file 3 — Reporting summary [file 41467_2024_51747_MOESM3_ESM.pdf]

nature portfolio

Corresponding author(s): Volker Spindler

Last updated by author(s): Jul 11, 2024

Reporting Summary

Nature Portfolio wishes to improve the reproducibility of the work that we publish. This form provides structure for consistency and transparency in reporting. For further information on Nature Portfolio policies, see our Editorial Policies and the Editorial Policy Checklist.

Statistics

For all statistical analyses, confirm that the following items are present in the figure legend, table legend, main text, or Methods section.

n/a	Confirmed	
☐	☒	The exact sample size (*n*) for each experimental group/condition, given as a discrete number and unit of measurement
☒	☐	A statement on whether measurements were taken from distinct samples or whether the same sample was measured repeatedly
☐	☒	The statistical test(s) used AND whether they are one- or two-sided<br>*Only common tests should be described solely by name; describe more complex techniques in the Methods section.*
☒	☐	A description of all covariates tested
☐	☒	A description of any assumptions or corrections, such as tests of normality and adjustment for multiple comparisons
☐	☒	A full description of the statistical parameters including central tendency (e.g. means) or other basic estimates (e.g. regression coefficient) AND variation (e.g. standard deviation) or associated estimates of uncertainty (e.g. confidence intervals)
☐	☒	For null hypothesis testing, the test statistic (e.g. F, t, r) with confidence intervals, effect sizes, degrees of freedom and P value noted<br>*Give P values as exact values whenever suitable.*
☒	☐	For Bayesian analysis, information on the choice of priors and Markov chain Monte Carlo settings
☒	☐	For hierarchical and complex designs, identification of the appropriate level for tests and full reporting of outcomes
☒	☐	Estimates of effect sizes (e.g. Cohen's d, Pearson's r), indicating how they were calculated

Our web collection on statistics for biologists contains articles on many of the points above.

Software and code

Policy information about availability of computer code

Data collection

LICOR odyssey, Image Studio Version 5.2 (Western Blots), INTAS GelStick IMAGER, Intas GDS Touch 2 (Gel development), Applied Biosystems StepOne Real-Time PCR System, StepOne Software v2.1 (RT-PCR), Hamamatsu NanoZoomerS60, NDPview 2 (Immunofluorescence scan), Stellaris 8 Falcon confocal microscope, LAS X acquisition and analysis software (Immunofluorescence), Integrative Genomics Viewer, Version 2.8.9 (ChIP-seq visualization), Synergy H1 microplate reader, Gen5 3.03 (BCA, HDAC activity, RNA concentration and quality), Thermo Scientific NanoDrop one and software (RNA, DNA concentration and quality), flow cytometry analyzer BC Cytoflex software (Flow cytometry), Q Exactive Plus mass spectrometer (Mass spectrometry)

Data analysis

CRISPR screen: raw sequencing reads were trimmed using Cutadapt with TTGTGGAAAGGACGAAACACCG as an adapter input with compulsory 22 overlap and allowing 2 indels or mismatches to remove the bases upstream of the sgRNA coding sequence including the variable stagger. Trimmed reads were aligned allowing for no mismatch with the human Brunello library using the count command of the MAGeCK software. Resulting read count tables were analyzed using the test command of MAGeCK by comparing the two biological replicates of Dsg3low over Dsg3high or vice versa. Hits were identified within the highest ranked genes that had a positive false discovery rate (FDR) < 0.2. For data analysis GraphPad Prism 8 was applied. Pictures were analyzed with Image J win64, NDPview 2 or QuPath-0.4.3. For flow cytometry FlowJo_v10.7.1 was used. For mass spectrometry data analysis MaxQuant version 1.6.50 was used.

For manuscripts utilizing custom algorithms or software that are central to the research but not yet described in published literature, software must be made available to editors and reviewers. We strongly encourage code deposition in a community repository (e.g. GitHub). See the Nature Portfolio guidelines for submitting code & software for further information.

Data

Policy information about availability of data

All manuscripts must include a data availability statement. This statement should provide the following information, where applicable:

- Accession codes, unique identifiers, or web links for publicly available datasets
- A description of any restrictions on data availability
- For clinical datasets or third party data, please ensure that the statement adheres to our policy

CRISPR screen data were deposited into the Gene Expression Omnibus database under accession number GSE244919 and are available at the following URL: https://www.ncbi.nlm.nih.gov/geo/query/acc.cgi?acc=GSE244919 (Reviewer token: sdaxkeasvxirrmx). All data supporting the findings of this study are available within the paper and its Supplementary Information.

Research involving human participants, their data, or biological material

Policy information about studies with human participants or human data. See also policy information about sex, gender (identity/presentation), and sexual orientation and race, ethnicity and racism.

Reporting on sex and gender	Ex vivo pemphigus skin model: Skin was obtained from cadavers of the body donor program of the Department of Anatomy Basel. The skin of male and female donors behaves in a similar way with regard to epidermal blister formation when treated with pemphigus vulgaris autoantibodies. Therefore, we did not analyze the skin samples according to sex or gender. The sex of the pemphigus vulgaris patients and controls is displayed in Supplementary Table 4.
Reporting on race, ethnicity, or other socially relevant groupings	n/a
Population characteristics	n/a
Recruitment	Tissue specimens were collected from volunteers who donated their bodies to the Institute of Anatomy for research and teaching after decease. Exclusion criteria were: Arrival > 16h after death and history or signs of infectious or skin diseases. Pemphigus vulgaris patient material was obtained from patients who gave written and informed consent in accordance with the local ethics committee (approved by the Ethics Commission of the Medical Faculty at the University of Marburg under the number 169/19). Production and cell culture of NHEK cells<br>Foreskin tissue was obtained during circumcision from patients who gave written and informed consent in accordance with the local ethics committee. (Ethikkommission Nordwest- und Zentralschweiz-EKNZ; date of approval: 11.06.2018, project ID: 2018-00963).
Ethics oversight	The experiments are carried out under the auspices of the University Basel Body Donor Program. The donors have given their written and informed consent for the use of tissue samples for teaching and research programs after their decease.

Note that full information on the approval of the study protocol must also be provided in the manuscript.

Field-specific reporting

Please select the one below that is the best fit for your research. If you are not sure, read the appropriate sections before making your selection.

☒ Life sciences ☐ Behavioural & social sciences ☐ Ecological, evolutionary & environmental sciences

For a reference copy of the document with all sections, see nature.com/documents/nr-reporting-summary-flat.pdf

Life sciences study design

All studies must disclose on these points even when the disclosure is negative.

Sample size	For animal studies, power analyses were conducted. No power analyses were performed for in vitro and ex vivo experiments. To ensure robustness and reproducibility, single experiments were repeated several times where indicated. The sample size varied between 3 and 8.
Data exclusions	Monolayers with more than 4 times the average fragmentation were excluded in dissociation assays. Data points that were significant outliers in the Grubbs test (GraphPad Prism 8) were excluded.
Replication	All experiments were repeated several times independently. The figure legend indicates the number of replicates for each sample
Randomization	Cell culture experiments (immunofluorescence, Western-blot, dissociation assays, RNA analyses, HDAC assays): All conditions being compared were performed on the same multi-well plate into which the cells were seeded to provide comparable growth conditions. Each condition was randomly distributed along the wells used and changed between experiments. Sample processing (homogenisation, centrifugation, RNA extraction, immunostaining) was randomised. Animal testing: Animals are randomly assigned to experimental groups, with each group given one specific treatment. Ex vivo skin and mucosa model: different treatments used skin or mucosa from the same donor site. Tissue processing was randomised.

Blinding	Researchers and technical personnel were not blinded to the test conditions.

Behavioural & social sciences study design

All studies must disclose on these points even when the disclosure is negative.

Study description	n/a
Research sample	n/a
Sampling strategy	n/a
Data collection	n/a
Timing	n/a
Data exclusions	n/a
Non-participation	n/a
Randomization	n/a

Ecological, evolutionary & environmental sciences study design

All studies must disclose on these points even when the disclosure is negative.

Study description	n/a
Research sample	n/a
Sampling strategy	n/a
Data collection	n/a
Timing and spatial scale	n/a
Data exclusions	n/a
Reproducibility	n/a
Randomization	n/a
Blinding	n/a

Did the study involve field work? ☐ Yes ☒ No

Field work, collection and transport

Field conditions	n/a
Location	n/a
Access & import/export	n/a
Disturbance	n/a

Reporting for specific materials, systems and methods

We require information from authors about some types of materials, experimental systems and methods used in many studies. Here, indicate whether each material, system or method listed is relevant to your study. If you are not sure if a list item applies to your research, read the appropriate section before selecting a response.

Materials & experimental systems

n/a	Involved in the study
☐	☒ Antibodies
☐	☒ Eukaryotic cell lines
☒	☐ Palaeontology and archaeology
☐	☒ Animals and other organisms
☐	☒ Clinical data
☒	☐ Dual use research of concern
☒	☐ Plants

Methods

n/a	Involved in the study
☒	☐ ChIP-seq
☐	☒ Flow cytometry
☒	☐ MRI-based neuroimaging

Antibodies

Antibodies used

KLF5 (Active Motif, #61099), HDAC3 (Abcam, #ab7030), GAPDH (Thermo Fisher Scientific, #16836913), H3ac (Lubioscience, #39040), DSG3 (Elab, #E-AB-62720), KLF5 K369ac (kindly provided by Jin-Tang Dong), anti-DSP-mAb (NW39), HDAC3 (Sigma, #HPA052052), AlexaFluor-coupled antibodies (Fisher Scientific, A-11008, A-11004), anti-HDAC3 (Abcam, ab137704), Goat anti-Mouse IRDye 800CW (LI-COR Biosciences, 925-32210), Goat anti-Rabbit IRDye 800CW (LI-COR Biosciences, 926-32211), Goat anti-Rabbit IRDye 800CW (LI-COR Biosciences, 925-68071), Normal Rabbit IgG (Cell Signaling Technology 2729S), PV-IgG (Dsg1 1207 U/ml und Dsg3 3906 U/ml) obtained from Enno Schmidt (Department of Dermatology, University of Lübeck), Ctrl-IgG were purified from approximately 30 ml blood plasma of control persons using agarose A resin (0.5 ml). Washing was performed with PBS, elution with 20mM citric acid pH 2.4.

Validation

The KLF5 (Active Motif, #61099) and HDAC3 (Abcam, #ab7030) antibodies were tested using sgRNA-mediated knockdown and/or overexpression experiments. All other antibodies listed were tested and validated by the manufacturers.

Eukaryotic cell lines

Policy information about cell lines and Sex and Gender in Research

Cell line source(s)

HaCaT were obtained from https://www.cytion.com/de/HaCaT-Zellen/300493. For NHEK isolation foreskin tissue was obtained during circumcision of patients after informed consent in accordance with the local ethics committee (EKNZ; date of approval: 11.06.2018, project ID: 2018-00963). The skin samples were washed three times in PBS containing 300 U/mL of penicillin (#A1837, AppliChem), 300 U/mL of streptomycin sulphate (#A1852, AppliChem) and 7.5 μg/mL of amphotericin B (#A2942 Sigma-Aldrich). The skin was cut into 0.5 x 1 cm pieces after removing excess tissue, blood vessels and parts of the dermis. For separation of dermis and epidermis, skin samples were immersed overnight at 4°C in 5 mg/mL Dispase II solution (#D4693, Sigma-Aldrich) in HBSS (#H8264, Sigma-Aldrich) containing 300 U/mL penicillin, 300 U/mL streptomycin sulphate and 2. 5 μg/mL amphotericin B. The epidermis was detached, washed once in PBS and digested in 0.25% trypsin and 1 mmol/L EDTA containing 100 U/mL penicillin and 100 U/mL streptomycin sulfate at 37°C for 20 minutes. The activity was stopped by a 1:1 dilution with a 1 mg/ml solution of soybean trypsinic inhibitor (#10684033, Gibco) in PBS. Keratinocytes were isolated by scraping epidermal debris from the bottom of the dish and passing through a 70µm cell filter (#431751, Corning, Somerville, USA). The isolated normal human epidermal keratinocytes (NHEK) were then seeded at a density of ~8 × 104 cells/cm2 in EpiLife medium containing 60 µmol/L CaCl2 (#MEPI500CA, Gibco) and 1% human keratinocyte supplement (#S0015, Gibco), 1% Pen/Strep and 2.5 µg/mL amphotericin B.

Authentication

Cell lines were routinely authenticated by STR profiling

Mycoplasma contamination

Cell lines were routinely tested for Mycoplasma contaminations.

Commonly misidentified lines
(See ICLAC register)

No commonly misidentified cell lines were used

Palaeontology and Archaeology

Specimen provenance

n/a

Specimen deposition

n/a

Dating methods

n/a

☐ Tick this box to confirm that the raw and calibrated dates are available in the paper or in Supplementary Information.

Ethics oversight

n/a

Note that full information on the approval of the study protocol must also be provided in the manuscript.

Animals and other research organisms

Policy information about studies involving animals; ARRIVE guidelines recommended for reporting animal research, and Sex and Gender in Research

Laboratory animals	BALB/c mice P1 (in vivo experiments)
Wild animals	n/a
Reporting on sex	Due to the age of the mice used, the reporting on sex is not applicable to animal experiments. For ex vivo experiments: Pemphigus is not prevalent in one sex/gender and autoantibodies show comparable effects in both sexes. In addition, no differences between male and female samples were observed in the morphology and composition of the epidermis. Therefore, no sex-specific data were obtained.
Field-collected samples	n/a
Ethics oversight	Veterinäramt Basel-Stadt (Number 3159)

Note that full information on the approval of the study protocol must also be provided in the manuscript.

Clinical data

Policy information about clinical studies

All manuscripts should comply with the ICMJE guidelines for publication of clinical research and a completed CONSORT checklist must be included with all submissions.

Clinical trial registration	n/a
Study protocol	n/a
Data collection	n/a
Outcomes	n/a

Dual use research of concern

Policy information about dual use research of concern

Hazards

Could the accidental, deliberate or reckless misuse of agents or technologies generated in the work, or the application of information presented in the manuscript, pose a threat to:

No	Yes	
☒	☐	Public health
☒	☐	National security
☒	☐	Crops and/or livestock
☒	☐	Ecosystems
☒	☐	Any other significant area

Experiments of concern

Does the work involve any of these experiments of concern:

No	Yes	
☒	☐	Demonstrate how to render a vaccine ineffective
☒	☐	Confer resistance to therapeutically useful antibiotics or antiviral agents
☒	☐	Enhance the virulence of a pathogen or render a nonpathogen virulent
☒	☐	Increase transmissibility of a pathogen
☒	☐	Alter the host range of a pathogen
☒	☐	Enable evasion of diagnostic/detection modalities
☒	☐	Enable the weaponization of a biological agent or toxin
☒	☐	Any other potentially harmful combination of experiments and agents

Plants

Seed stocks	n/a
Novel plant genotypes	n/a
Authentication	n/a

ChIP-seq

Data deposition

☐ Confirm that both raw and final processed data have been deposited in a public database such as GEO.

☐ Confirm that you have deposited or provided access to graph files (e.g. BED files) for the called peaks.

Data access links<br>*May remain private before publication.*	*For "Initial submission" or "Revised version" documents, provide reviewer access links. For your "Final submission" document, provide a link to the deposited data.*
Files in database submission	*Provide a list of all files available in the database submission.*
Genome browser session<br>(e.g. UCSC)	*Provide a link to an anonymized genome browser session for "Initial submission" and "Revised version" documents only, to enable peer review. Write "no longer applicable" for "Final submission" documents.*

Methodology

Replicates	*Describe the experimental replicates, specifying number, type and replicate agreement.*
Sequencing depth	*Describe the sequencing depth for each experiment, providing the total number of reads, uniquely mapped reads, length of reads and whether they were paired- or single-end.*
Antibodies	*Describe the antibodies used for the ChIP-seq experiments; as applicable, provide supplier name, catalog number, clone name, and lot number.*
Peak calling parameters	*Specify the command line program and parameters used for read mapping and peak calling, including the ChIP, control and index files used.*
Data quality	*Describe the methods used to ensure data quality in full detail, including how many peaks are at FDR 5% and above 5-fold enrichment.*
Software	*Describe the software used to collect and analyze the ChIP-seq data. For custom code that has been deposited into a community repository, provide accession details.*

Flow Cytometry

Plots

Confirm that:

☒ The axis labels state the marker and fluorochrome used (e.g. CD4-FITC).

☒ The axis scales are clearly visible. Include numbers along axes only for bottom left plot of group (a 'group' is an analysis of identical markers).

☒ All plots are contour plots with outliers or pseudocolor plots.

☒ A numerical value for number of cells or percentage (with statistics) is provided.

Methodology

Sample preparation	For FCM, HaCaT cells were harvested with Trypsin (Thermo Fisher scientific, #T/3760/48) and transferred to a 96-well plate. Cells were washed with FACS buffer (PBS, 2% FCS, 0,1% Sodium azide) and incubated with anti-DSG3-647 (1:200; Santa Crus Biotechnology, #sc-53487) and Zombie Aqua (1:200, BioLegend, #423101) for 30 min at 4°C. Stained cells were washed with FACS buffer once, fixed with 4% Paraformaldehyde (in PBS, Fisher Scientific, #10131580) washed with FACS buffer and resuspended in FACS buffer. Staining of the cells was measured using flow cytometry analyzer BC Cytoflex.

Instrument	BC Cytoflex
Software	Flow Jo 10.7.1
Cell population abundance	Cell population abundance is detailed in Supplementary Fig. 1a.
Gating strategy	The cell were plotted in forward and side scatter (FSC, SSC) and cells below 50 were excluded as cell debris. Cells positive for Zombiaqua were excludes as dead cells. The gating for cells positive for GFP, BPF is detailed in Supplementary Fig. 1a.

☒ Tick this box to confirm that a figure exemplifying the gating strategy is provided in the Supplementary Information.

Magnetic resonance imaging

Experimental design

Design type	*Indicate task or resting state; event-related or block design.*
Design specifications	*Specify the number of blocks, trials or experimental units per session and/or subject, and specify the length of each trial or block (if trials are blocked) and interval between trials.*
Behavioral performance measures	*State number and/or type of variables recorded (e.g. correct button press, response time) and what statistics were used to establish that the subjects were performing the task as expected (e.g. mean, range, and/or standard deviation across subjects).*

Acquisition

Imaging type(s)	*Specify: functional, structural, diffusion, perfusion.*
Field strength	*Specify in Tesla*
Sequence & imaging parameters	*Specify the pulse sequence type (gradient echo, spin echo, etc.), imaging type (EPI, spiral, etc.), field of view, matrix size, slice thickness, orientation and TE/TR/flip angle.*
Area of acquisition	*State whether a whole brain scan was used OR define the area of acquisition, describing how the region was determined.*

Diffusion MRI ☐ Used ☐ Not used

Preprocessing

Preprocessing software	*Provide detail on software version and revision number and on specific parameters (model/functions, brain extraction, segmentation, smoothing kernel size, etc.).*
Normalization	*If data were normalized/standardized, describe the approach(es): specify linear or non-linear and define image types used for transformation OR indicate that data were not normalized and explain rationale for lack of normalization.*
Normalization template	*Describe the template used for normalization/transformation, specifying subject space or group standardized space (e.g. original Talairach, MNI305, ICBM152) OR indicate that the data were not normalized.*
Noise and artifact removal	*Describe your procedure(s) for artifact and structured noise removal, specifying motion parameters, tissue signals and physiological signals (heart rate, respiration).*
Volume censoring	*Define your software and/or method and criteria for volume censoring, and state the extent of such censoring.*

Statistical modeling & inference

Model type and settings	*Specify type (mass univariate, multivariate, RSA, predictive, etc.) and describe essential details of the model at the first and second levels (e.g. fixed, random or mixed effects; drift or auto-correlation).*
Effect(s) tested	*Define precise effect in terms of the task or stimulus conditions instead of psychological concepts and indicate whether ANOVA or factorial designs were used.*

Specify type of analysis: ☐ Whole brain ☐ ROI-based ☐ Both

Statistic type for inference (See Eklund et al. 2016)	*Specify voxel-wise or cluster-wise and report all relevant parameters for cluster-wise methods.*
Correction	*Describe the type of correction and how it is obtained for multiple comparisons (e.g. FWE, FDR, permutation or Monte Carlo).*

Models & analysis

n/a	Involved in the study
☐	☐ Functional and/or effective connectivity
☐	☐ Graph analysis
☐	☐ Multivariate modeling or predictive analysis

Functional and/or effective connectivity	*Report the measures of dependence used and the model details (e.g. Pearson correlation, partial correlation, mutual information).*
Graph analysis	*Report the dependent variable and connectivity measure, specifying weighted graph or binarized graph, subject- or group-level, and the global and/or node summaries used (e.g. clustering coefficient, efficiency, etc.).*
Multivariate modeling and predictive analysis	*Specify independent variables, features extraction and dimension reduction, model, training and evaluation metrics.*
